# The association between economic uncertainty and suicide in Japan by age, sex, employment status, and population density: an observational study

**DOI:** 10.1016/j.lanwpc.2024.101069

**Published:** 2024-05-02

**Authors:** Haruka Goto, Ichiro Kawachi, Sotiris Vandoros

**Affiliations:** a University College London (UCL), London, United Kingdom; b Harvard T.H. Chan School of Public Health, Boston, United States

**Keywords:** Suicide, Mental health, Economic uncertainty, Japan

## Abstract

**Background:**

Suicide is one of the ten leading causes of death globally, and previous research has revealed a link between economic conditions and mental health. However, the literature has focused primarily on recessions and unemployment, i.e. actual economic developments, as opposed to uncertainty, which relates to economic developments that have not (yet) materialised. This study examines the differential association between economic uncertainty and suicide in Japan, depending on age, sex, employment status, and population density, in order to identify the groups that are affected the most.

**Methods:**

Using monthly prefectural suicide mortality data from the Ministry of Health, Labour and Welfare and a monthly economic uncertainty index for the period 2009 to 2019, we employed a fixed effects panel data approach to examine the association between uncertainty and suicide by population group.

**Findings:**

We found that a 1% increase in economic uncertainty is associated with a 0.061 increase in the monthly number of suicides per 100,000 people per prefecture, on average (coefficient: 6.08; 95% CI: 5.07–7.08), which constitutes a 3.62% increase. Self-employed people, as well as men in their 50s and unemployed men, experience the highest additional risk of suicide when uncertainty increases. The association was approximately three times stronger for males than for females, and a strong association was observed for self-employed males living in more densely-populated areas.

**Interpretation:**

Uncertainty appears to relate to suicides for most groups, but self-employed people, males, and those living in more densely populated areas appear to be more at risk of suicide in periods of increased economic uncertainty. Our results provide an indication of which groups mental health services and prevention strategies can focus on in times of economic uncertainty.

**Funding:**

None.

**Research in context**

**Evidence before this study**

The literature has focused on the relationship between current economic conditions (such as unemployment) on suicide. Some studies argue that economic uncertainty (rather than actual economic developments) appears to be associated with suicide, but there is no evidence on which population groups might be affected the most.

**Added value of this study**

This study employs a panel data approach to study the association between economic uncertainty and suicide in Japan, providing evidence by age, sex, employment status, and population density. This paper thus contributes to the literature by providing evidence on the association between economic uncertainty and suicide (a) by population group (age, sex, working status, and population density), and (b) by being the first to study this association in Japan. We found a positive relationship between economic uncertainty and suicide. Our results show that self-employed people, as well as men in their 50s and unemployed men, experience the highest additional risk of suicide when uncertainty increases. The association was approximately three times stronger for males than for females, and a strong association was observed for self-employed males living in more densely-populated areas.

**Implications of all the available evidence**

Our results provide an indication of which groups mental health services and prevention strategies can focus on in times of economic uncertainty. Future research can examine the role of education on the association between uncertainty and suicide.

## Introduction

More than 700,000 people die from suicide globally every year,^1^ making suicide prevention and the understanding of the causes of suicide an important part of public health research. In Japan, 21,007 people lost their lives to suicide in 2021, with a death rate of 16.7 per 100,000 people.^2^ Based on the World Health Organisation's estimate of age-standardised suicide rates, Japan recorded a rate of 12.2 suicides per 100,000 people in 2019, surpassing the global age-standardised suicide rate of 9.0 in the same year.^3^ The Japanese Government considers suicide prevention to be an important issue, as evidenced by its budget request of more than JPY 3.8 billion for the promotion of suicide prevention in 2022.^4^ Similarly, other nations prioritise this critical issue. The UK allocated £2.3 billion for mental health services, earmarking £57 million specifically for suicide prevention by March 2024.^5^ In the US, the National Centre for Injury Prevention and Control (NCIPC) also boosted its FY2022 budget for suicide prevention to $20 million, compared to $10 million in FY2020.^6^

External factors can often play a significant role when it comes to the causes of suicide. The literature presents a plethora of evidence on the link between economic conditions and suicide.^7^, ^8^, ^9^, ^10^, ^11^ The positive correlation between the suicide rate and unemployment^10^ or economic crises^11^ has been observed in 21 OECD countries. This association is also evident and stronger in Japan compared to other OECD countries, as reflected by data from 1980 to 2000, when suicide rates were increasing.^12^ Even between 2009 and 2018, a period of declining suicide rates in Japan, the link between suicide and unemployment persisted among older males in the country.^13^ Despite the robust evidence between suicide and unemployment, previous studies have demonstrated that suicide mortality might exhibit a non-linear and asymmetrical response to unemployment during economic crises.^10^^,^^14^, ^15^, ^16^ This suggests the necessity for a more thorough disaggregation and investigation of external factors.

**Fig. 1:**
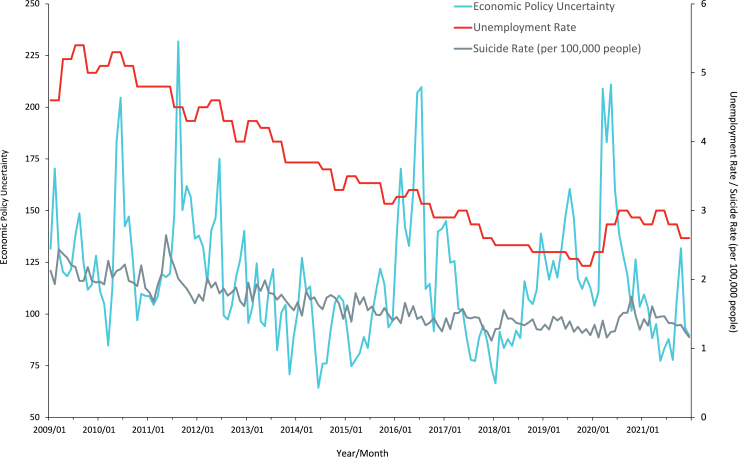
**Trends in suicide rates, unemployment rates and the economic policy uncertainty index**. The possible explanations for increased the economic policy uncertainty: May–June 2010: Greek crisis prompts heightened uncertainty. PM Hatoyama resigns; PM Kan assumes office, leading to a new cabinet installation./August 2011: U.S. debt-ceiling crisis and concerns over the European debt crisis contribute to uncertainty. PM Kan resigns amidst further monetary easing and Japan's FX intervention./May–June 2016: Uncertainty rises due to the delay in the consumption tax hike and the Brexit referendum./November 2016–January 2017: Economic policy uncertainty escalates with the U.S. Presidential election and withdrawal from the TPP./March 2020: The Covid-19 outbreak in Japan sparks debates on fiscal stimulus and monetary easing, intensifying economic policy uncertainty.^17^

While recessions and unemployment represent economic circumstances that have already unfolded, economic uncertainty relates to economic developments that have yet to occur (or may not even materialise at all). In fact, although economic downturns sometimes coincide with increased uncertainty, this is not always the case. Fig. 1 shows that uncertainty and unemployment have different trends in Japan. A similar example was the post-Brexit period in the UK that was characterised by increased uncertainty despite low levels of unemployment.^17^ The aftermath of the Trump election in the US was also a similar situation.^17^ The association between uncertainty and suicides therefore merits investigation, over and above existing studies on recessions/unemployment and suicides.

Studies on the health effects of uncertainty have only recently begun attracting attention in the literature. There is a positive association between economic uncertainty and suicide^18^, ^19^, ^20^, ^21^, ^22^, ^23^ and the association appears to be stronger for males aged 25–64 and females aged over 65 in the US.^21^ A study on 183 countries also revealed a delayed effect of global economic uncertainty on suicide rates.^22^ Apart from suicide, uncertainty also appears to be associated with cardiovascular mortality^15^^,^^24^ and car crashes, possibly due to distraction, frustration, and sleep deprivation.^25^ Events triggering uncertainty at the national level also appear to be associated with people's mood and well-being,^26^, ^27^, ^28^ and a recent study demonstrated a negative correlation between economic uncertainty and subjective health among men aged 25–54 and women under 25.^29^

Despite growing evidence, several gaps remain in our understanding of the association between economic uncertainty and suicide. First, the extent of the association between uncertainty and suicide may depend on employment status as self-employed people are often more vulnerable to suicide compared to employees.^30^ Second, potential heterogeneity by region needs to be understood, as there might be different characteristics of suicide between local regions.^31^ Third, there is a need to focus on a more diverse group of countries. Most existing studies focus primarily on Western countries, and in particular on the US and the UK.^18^, ^19^, ^20^ While some papers examined the correlation between global economic uncertainty and suicide by including Japan in the ‘East Asia’^22^ and ‘high-income’ group,^23^ another study has emphasised the considerable heterogeneity in the relationship between economic uncertainty and health across countries,^32^ suggesting the need for country-specific studies.

The objective of this study is to examine the association between suicide and economic uncertainty by age, sex, employment status, and population density in Japan. While recent studies provided evidence on the association between uncertainty and suicide in total, there was no evidence on how uncertainty relates to different population groups. This paper thus contributes to the literature by providing detailed evidence on the association between economic uncertainty and suicide (a) by age, sex, working status, and population density, and (b) by being the first to study this association particularly in Japan. It thus provides evidence on within-country heterogeneity, which can help develop further policies targeting vulnerable groups in unpredictable future instability.

## Methods

The main variable of interest is the monthly prefectural suicide rate, measured as the number of suicides per 100,000 people. Data between January 2009 and December 2019 were obtained from the Basic Data on Suicide in the Region (BDSR) database, provided by the Ministry of Health, Labour and Welfare of Japan.^33^ The BDSR dataset includes information on employment status and is reported at the prefecture level (Japan has 47 prefectures), and we also used suicide rates by sex, age group, population density, and occupation. Monthly suicides by prefecture are recorded as missing if the number of suicides is one or two per population group, to ensure anonymity. However, this omitted observation issue is less severe than it would have been if we had used US data on suicides, which excludes observations with fewer than 10 suicides, as used in previous studies.^20^^,^^34^ The suicide rate was calculated based on prefecture-specific population estimates,^35^ but, specific population data by occupation were only accessible through the census that takes place every five years.^36^^,^^37^ To address this, we employed linear extrapolation to estimate missing data, utilising population figures from the 2005, 2010, 2015, and 2020 census years.

We used the monthly Japan Economic Policy Uncertainty (EPU) index^38^ for the period 2009–2019 to capture economic uncertainty, in line with previous studies.^15^^,^^18^, ^19^, ^20^, ^21^, ^22^, ^23^, ^24^, ^25^^,^^29^^,^^32^ This index was developed to capture uncertainty in Japan using key terms from four major Japanese newspapers: Yomiuri, Asahi, Mainichi, and Nikkei.^17^ As in previous research on economic policy uncertainty in other countries,^39^ terms related to the economy, uncertainty, and policy were collected from these four newspapers. The collected terms were scaled based on the number of articles published in the same newspaper and the same month. Subsequently, to calculate the EPU index, the article counts were standardised, adjusted for seasonality, and then averaged across articles on a monthly basis. A notable peak in the EPU index in Japan coincided with events such as major leadership changes and the financial crisis (Fig. 1). Furthermore, the index exhibits a positive correlation with various indicators, including the implied volatility of stocks, exchange rates, and interest rates.^17^

We also collected data on unemployment rates at the prefecture level, obtained from the Labour Force Survey, provided by the Statistics Bureau of Japan.^40^ The quarterly average unemployment rates per prefecture from January 2009 to December 2019 were applied to the unemployment rate for each corresponding month and prefecture since the unemployment rate by prefecture is only published as quarterly averages. Fig. 1 shows trends in suicides, unemployment, and the economic uncertainty index. Despite observing a correlation between the EPU index and the unemployment rate (Table A1), these two variables do not coincide, nor do they always move in the same direction, and notable gaps are observed between the peaks of the EPU index and the unemployment rate.

Furthermore, we used population density data from the Population Census in 2015 to examine urban-rural differences, captured by population density.^35^ Population density is used because it has been shown to be one of the most sensitive proxy indicators for modelling urban-rural variation in suicide mortality.^41^ The average population density in Japan was 655.3/km^2^. The 8 prefectures where density exceeded this figure (Tokyo, Osaka, Kanagawa, Saitama, Aichi, Chiba, Fukuoka, and Hyogo), were considered densely-populated areas, and the other 39 prefectures were considered low-population density areas.

We followed a panel data approach using Stata version 17 to provide a comprehensive understanding of the association between economic uncertainty and suicide rates while accounting for time series and individual prefectures’ characteristics. Specifically, we adopted a fixed effects model to control for all variables that vary across prefectures but are constant over time, thereby accounting for unobserved heterogeneity and reducing the possibility of omitted variable bias.^42^ The dependent variable is the suicide rate, and the main explanatory variable is the uncertainty index. We controlled for the local unemployment rate to rule out that any association might be driven by actual economic conditions rather than uncertainty alone. Year-month dummies were included as control variables to account for seasonality and annual trends. Augmented-Dickey-Fuller tests were conducted to check the unit-roots of the data, and the null hypothesis was rejected for the unit roots for suicide rate and economic uncertainty. The fixed effects model is presented in Equation (1)

(1) $${Suicide\;Rate}_{it}=\alpha_{i}\;+\;\beta_{1}\;\ln\;{uncertainty}_{t}+\;\beta_{2}\;u{nemployment\;rate}_{it}\;+\;\tau_{t}\;+\;\varepsilon_{it}$$

The suffixes (*i* and *t*) denote the prefecture and time, respectively, and the dependent variable *Suicide Rate*_*it*_ denotes the number of suicides per 100,000 people in the local prefecture *i* and time *t.* The model term α represents the fixed effect for each prefecture *i*. The explanatory variable, *uncertainty*, is the natural logarithm of the monthly economic uncertainty index. Applying the natural logarithm of the uncertainty index in the model enhances interpretability by stabilising variable variance, addressing concerns related to heteroscedasticity, and normalising the distribution of the variable. Control variable $u{nemployment\;rate}_{it}$, captures the quarterly unemployment rate of each prefecture, $\tau_{t}$ represents the year-month dummy variables, and $\varepsilon_{it}$ is the error term.

We also investigated the potential non-linearity in the association between economic uncertainty and suicide rate. Following previous research,^15^ the association of EPU was controlled by dividing the EPU into three parts and introducing dummy variables for the bottom EPU and top EPU. In this context, $Top\;{uncertainty}_{t}$ and $Bottom\;uncertainty_{t}$ denote dummy variables corresponding to the upper and lower thirds (terciles) of the EPU distribution, respectively.

Apart from the baseline model, we stratified by age group, sex, employment status (employed, self-employed, unemployed, student), and population density. Moreover, an interaction term between economic uncertainty and the unemployment rate was introduced to examine the EPU and each prefecture's unemployment rate jointly, potentially increasing the risk of suicide. Finally, we examined the association between lagged EPU and suicide.

## Results

Summary statistics are presented in Table A2 in the Online Appendix. The average monthly suicide mortality per prefecture is 41.97 (SD 41.24), with male suicides accounting for more than two-thirds of this total. Prefectures characterised by lower population density demonstrate higher suicide rates. Examining employment status, the highest average monthly suicide mortality per prefecture is observed among unemployed individuals at 3.21 per 100,000 people, followed by self-employed persons at 2.19, employed persons at 1.21, and students at 0.42.

Results of the baseline panel data model, in total and by sex and population density, are presented in Table 1. There is a strong positive and statistically significant correlation between the natural logarithm of economic uncertainty and the suicide rate in all nine regressions. Results of the model that shows results for the entire population are presented in Column 1. Overall, a 1% increase in economic uncertainty is associated with a 0.061 (coefficient: 6.08; 95% CI: 5.07–7.08) increase in the monthly number of suicides per 100,000 people per prefecture, on average–a 3.62% increase. When running separate regressions by sex (Columns 2-3), the association is stronger for males (coefficient: 9.36; 95% CI: 7.62–11.11) than for females (coefficient: 3.04; 95% CI: 2.03–4.05), but positive and significant in both cases. Columns (4) and (7) present the results for prefectures with high and low population density, respectively. In both cases, the association is positive and significant, but the coefficient is higher in areas with lower population density (6.21) compared to those with higher density (5.01). Columns (5–6) and (8–9) show results by sex and density, which confirm that the association is stronger for males than for females. Interestingly, of these nine regressions, the unemployment rate appears to be significant only when considering the total or male population in high-density areas (Columns 4–5)—suggesting that it might be economic uncertainty primarily driving any association between economic conditions and health, rather than unemployment per se.

**Table 1:** Results of the baseline fixed effects model by sex and population density.

	Whole			Urban			Rural		
	Total	Male	Female	Total	Male	Female	Total	Male	Female
Dependent Variable: Suicides per 100,000 people	(1)	(2)	(3)	(4)	(5)	(6)	(7)	(8)	(9)
Uncertainty	6.08∗∗∗	9.36∗∗∗	3.04∗∗∗	5.01∗∗∗	7.40∗∗∗	2.58∗∗∗	6.21∗∗∗	9.63∗∗∗	3.09∗∗∗
	(5.07 to 7.08)	(7.62 to 11.11)	(2.03 to 4.05)	(3.86 to 6.17)	(5.48 to 9.31)	(1.40 to 3.76)	(5.02 to 7.40)	(7.57 to 11.70)	(1.90 to 4.28)
Unemployment rate	0.02	0.03	0.01	0.07∗∗∗	0.12∗∗∗	0.02	0.02	0.03	0.01
	(−0.00 to 0.04)	(−0.01 to 0.08)	(−0.02 to 0.03)	(0.03 to 0.10)	(0.06 to 0.18)	(−0.01 to 0.06)	(−0.01 to 0.05)	(−0.02 to 0.08)	(−0.02 to 0.04)
Constant	−27.58∗∗∗	−42.62∗∗∗	−13.69∗∗∗	−22.74∗∗∗	−33.76∗∗∗	−11.47∗∗∗	−28.20∗∗∗	−43.83∗∗∗	−13.96∗∗∗
	(−32.37 to −22.79)	(−50.93 to −34.31)	(−18.50 to −8.88)	(−28.22 to −17.27)	(−42.87 to −24.65)	(−17.09 to −5.86)	(−33.87 to −22.53)	(−53.67 to −33.99)	(−19.65 to −8.26)
Observations	6204	6204	6204	1056	1056	1056	5148	5148	5148
N of Prefectures	47	47	47	8	8	8	39	39	39
Adjusted R-squared	0.466	0.395	0.208	0.772	0.716	0.536	0.439	0.373	0.187
F-value	42.33	32.08	13.66	28.08	21.22	10.29	31.85	24.51	10.23
Degrees of Freedom	6025	6025	6025	916	916	916	4977	4977	4977

Notes: Fixed Effects Panel Data. Uncertainty in natural logarithm. Year-month dummies included. Confidence intervals in parentheses. ∗∗∗p < 0.01, ∗∗p < 0.05, ∗p < 0.1.

The regression results by age group are detailed in Table 2. For individuals aged 10–19, there is no discernible association between economic uncertainty and suicides (Columns 1–3). Among those aged 20–29, a positive association is evident (Columns 4–6), but it is insignificant for females (Column 6). In the 30s and 40s age group, the relationships are positive and significant for both sexes (Columns 7–9), but the evidence of the association is weak among females in their 40s (Columns 10–12). The strongest association by age groups is present for men in their 50s (Column 14), yet no correlation is observed for females in the same age group (Column 15). For individuals aged over 60, a consistently positive and significant association is evident for both males and females, with the exception of females aged 80 years and over (Columns 16–24).

**Table 2:** Results by Age group.

	10–19			20–29			30–39			40–49		
	Total	Male	Female	Total	Male	Female	Total	Male	Female	Total	Male	Female
Dependent Variable: Suicides per 100,000 people	(1)	(2)	(3)	(4)	(5)	(6)	(7)	(8)	(9)	(10)	(11)	(12)
Uncertainty	0.27	0.79	−0.30	6.12∗∗∗	9.28∗∗∗	2.64	5.63∗∗∗	6.79∗∗∗	4.62∗∗∗	5.20∗∗∗	8.01∗∗∗	2.48∗
	(−0.50 to 1.04)	(−0.44 to 2.02)	(−1.21 to 0.61)	(2.81 to 9.43)	(3.74 to 14.83)	(−0.75 to 6.04)	(2.74 to 8.53)	(1.79 to 11.79)	(1.75 to 7.49)	(2.27 to 8.12)	(2.84 to 13.18)	(−0.35 to 5.32)
Unemployment rate	0.00	−0.01	0.02∗	−0.03	−0.12∗	0.06	0.03	0.03	0.02	0.07∗	0.11∗	0.04
	(−0.01 to 0.02)	(−0.04 to 0.02)	(−0.00 to 0.04)	(−0.11 to 0.05)	(−0.25 to 0.02)	(−0.02 to 0.15)	(−0.05 to 0.10)	(−0.09 to 0.15)	(−0.05 to 0.09)	(−0.00 to 0.14)	(−0.02 to 0.23)	(−0.03 to 0.11)
Constant	−1.10	−3.45	1.47	−27.64∗∗∗	−42.04∗∗∗	−11.80	−25.30∗∗∗	−30.13∗∗	−21.20∗∗∗	−23.03∗∗∗	−35.55∗∗∗	−11.02
	(−4.77 to 2.56)	(−9.33 to 2.43)	(−2.87 to 5.81)	(−43.44 to −11.85)	(−68.50 to −15.58)	(−27.98 to 4.39)	(−39.12 to −11.48)	(−54.00 to −6.26)	(−34.90 to −7.51)	(−36.97 to −9.08)	(−60.23 to −10.88)	(−24.53 to 2.48)
Observations	6204	6201	6165	6204	6201	6165	6204	6201	6165	6204	6201	6165
N of Prefectures	47	47	47	47	47	47	47	47	47	47	47	47
Adjusted R-squared	0.006	0.000	−0.005	0.050	0.031	0.019	0.088	0.056	0.038	0.157	0.135	0.027
F-value	1.639	1.360	1.127	3.816	2.837	2.246	5.883	4.147	3.191	10.12	8.650	2.632
Degrees of Freedom	6025	6022	5986	6025	6022	5986	6025	6022	5986	6025	6022	5986

	**50–59**			**60–69**			**70–79**			**80-**		
	Total	Male	Female	Total	Male	Female	Total	Male	Female	Total	Male	Female
Dependent Variable: Suicides per 100,000 people	(13)	(14)	(15)	(16)	(17)	(18)	(19)	(20)	(21)	(22)	(23)	(24)
Uncertainty	11.43∗∗∗	20.37∗∗∗	2.50	8.85∗∗∗	11.96∗∗∗	5.91∗∗∗	6.33∗∗∗	8.68∗∗∗	4.61∗∗∗	8.17∗∗∗	18.04∗∗∗	3.35∗
	(8.25 to 14.62)	(14.75 to 25.99)	(−0.59 to 5.60)	(6.10 to 11.61)	(7.19 to 16.72)	(3.04 to 8.78)	(3.14 to 9.51)	(3.06 to 14.30)	(1.10 to 8.11)	(4.46 to 11.88)	(9.86 to 26.23)	(−0.34 to 7.04)
Unemployment rate	0.08∗∗	0.16∗∗	0.01	0.05	0.12∗∗	−0.01	0.04	0.10	−0.00	−0.10∗∗	−0.24∗∗	−0.02
	(0.01 to 0.16)	(0.03 to 0.30)	(−0.07 to 0.08)	(−0.02 to 0.12)	(0.01 to 0.24)	(−0.08 to 0.06)	(−0.04 to 0.12)	(−0.04 to 0.24)	(−0.09 to 0.08)	(−0.19 to −0.01)	(−0.44 to −0.04)	(−0.11 to 0.07)
Constant	−52.68∗∗∗	−94.45∗∗∗	−10.92	−40.73∗∗∗	−54.82∗∗∗	−27.39∗∗∗	−28.82∗∗∗	−39.57∗∗∗	−21.01∗∗	−37.01∗∗∗	−82.62∗∗∗	−14.81∗
	(−67.86 to −37.50)	(−121.25 to −67.65)	(−25.68 to 3.85)	(−53.87 to −27.58)	(−77.56 to −32.09)	(−41.08 to −13.70)	(−44.01 to −13.62)	(−66.39 to −12.74)	(−37.71 to −4.30)	(−54.70 to −19.31)	(−121.68 to −43.56)	(−32.41 to 2.80)
Observations	6204	6201	6165	6204	6201	6165	6204	6201	6165	6204	6201	6165
N of Prefectures	47	47	47	47	47	47	47	47	47	47	47	47
Adjusted R-squared	0.193	0.186	0.015	0.204	0.168	0.051	0.111	0.068	0.047	0.087	0.037	0.058
F-value	12.60	12.08	2.049	13.40	10.86	3.844	7.190	4.776	3.669	5.838	3.166	4.241
Degrees of Freedom	6025	6022	5986	6025	6022	5986	6025	6022	5986	6025	6022	5986

Notes: Fixed Effects Panel Data. Uncertainty in natural logarithm. Year-month dummies included. Confidence intervals in parentheses. ∗∗∗p < 0.01, ∗∗p < 0.05, ∗p < 0.1.

Results of regressions by working status are presented in Table 3. With regards to self-employed people (Columns 1–3), economic uncertainty is positively associated with suicides overall (Column 1). The association is significant for both males and females (Columns 2–3). The association among employed individuals is also positive and significant overall (Column 4), as well as for males (Column 5), but not for females (Column 6). Similar results hold for unemployed people (Columns 7–9), and a positive and significant association is present for the total population (Column 7) and unemployed males (Column 8), but no evidence for unemployed females (Column 9). Student suicide does not appear to be correlated with economic uncertainty (Columns 10–12).

**Table 3:** Results by occupation.

	Self-employed			Employed			Unemployed			Student		
	Total	Male	Female	Total	Male	Female	Total	Male	Female	Total	Male	Female
Dependent Variable: Suicides per 100,000 people	(1)	(2)	(3)	(4)	(5)	(6)	(7)	(8)	(9)	(10)	(11)	(12)
Uncertainty	9.36∗∗∗	13.94∗∗∗	5.04∗∗∗	3.63∗∗∗	5.12∗∗∗	0.68	25.11∗∗∗	29.39∗∗∗	3.62	1.00	1.29	0.23
	(5.29 to 13.44)	(7.33 to 20.54)	(1.39 to 8.68)	(2.30 to 4.96)	(2.84 to 7.41)	(−0.60 to 1.95)	(15.71 to 34.51)	(15.75 to 43.04)	(−5.42 to 12.66)	(−0.34 to 2.35)	(−0.98 to 3.55)	(−1.34 to 1.79)
Unemployment rate	0.04	0.01	0.00	0.04∗∗	0.08∗∗∗	0.02	−0.31∗∗∗	−0.32∗	−0.05	−0.00	0.00	0.01
	(−0.06 to 0.14)	(−0.14 to 0.17)	(−0.09 to 0.09)	(0.01 to 0.07)	(0.02 to 0.13)	(−0.01 to 0.05)	(−0.54 to −0.09)	(−0.65 to 0.00)	(−0.26 to 0.17)	(−0.04 to 0.03)	(−0.05 to 0.06)	(−0.02 to 0.05)
Constant	−42.91∗∗∗	−63.74∗∗∗	−23.69∗∗∗	−16.21∗∗∗	−22.83∗∗∗	−2.78	−115.98∗∗∗	−135.41∗∗∗	−15.28	−4.40	−5.58	−0.92
	(−62.36 to −23.47)	(−95.29 to −32.18)	(−41.13 to −6.25)	(−22.54 to −9.89)	(−33.73 to −11.93)	(−8.89 to 3.34)	(−160.82 to −71.15)	(−200.61 to −70.22)	(−58.47 to 27.92)	(−10.82 to 2.03)	(−16.41 to 5.25)	(−8.40 to 6.55)
Observations	6196	5760	5759	6196	5760	5759	6196	5760	5759	6196	5760	5759
N of Prefectures	47	47	47	47	47	47	47	47	47	47	47	47
Adjusted R-squared	0.127	0.130	−0.004	0.177	0.156	0.020	0.065	0.066	−0.001	0.008	0.005	−0.003
F-value	8.185	7.895	1.170	11.42	9.397	2.242	4.631	4.452	1.302	1.733	1.554	1.240
Degrees of Freedom	6017	5581	5580	6017	5581	5580	6017	5581	5580	6017	5581	5580

Notes: Fixed Effects Panel Data. Uncertainty in natural logarithm. Year-month dummies included. Confidence intervals in parentheses. ∗∗∗p < 0.01, ∗∗p < 0.05, ∗p < 0.1.

Table 4 presents results of the non-linear model. Results suggest that periods of high uncertainty were positively associated with suicide mortality (Columns 1–3), while periods of low uncertainty demonstrated a negative association with suicide (Columns 4–6).

**Table 4:** Results of the non-linear fixed-effects model by sex.

	Top terciles of uncertainty			Bottom terciles of uncertainty		
	Total	Male	Female	Total	Male	Female
Dependent Variable: Suicides per 100,000 people	(1)	(2)	(3)	(4)	(5)	(6)
Uncertainty	0.94∗∗∗	1.45∗∗∗	0.47∗∗∗	−0.71∗∗∗	−1.10∗∗∗	−0.34∗∗∗
	(0.79 to 1.10)	(1.18 to 1.71)	(0.31 to 0.63)	(−0.86 to −0.55)	(−1.37 to −0.84)	(−0.49 to −0.18)
Unemployment rate	0.02	0.03	0.01	0.02	0.03	0.01
	(−0.00 to 0.04)	(−0.01 to 0.08)	(−0.02 to 0.03)	(−0.00 to 0.04)	(−0.01 to 0.08)	(−0.02 to 0.03)
Constant	1.13∗∗∗	1.63∗∗∗	0.66∗∗∗	2.07∗∗∗	3.08∗∗∗	1.13∗∗∗
	(1.02 to 1.24)	(1.44 to 1.82)	(0.55 to 0.77)	(1.92 to 2.22)	(2.82 to 3.34)	(0.98 to 1.28)
Observations	6204	6204	6204	6204	6204	6204
N of Prefectures	47	47	47	47	47	47
Adjusted R-squared	0.466	0.395	0.208	0.466	0.395	0.208
F-value	42.33	32.08	13.66	42.33	32.08	13.66
Degrees of Freedom	6025	6025	6025	6025	6025	6025

Notes: Fixed Effects Panel Data. Uncertainty in natural logarithm. Year-month dummies included. Confidence intervals in parentheses. ∗∗∗p < 0.01, ∗∗p < 0.05, ∗p < 0.1.

Results presented in Table A3 in the Online Appendix demonstrate that the interaction term between uncertainty and suicide is positive and statistically significant, although the coefficient is small. Table A4 illustrates the relationship between lagged EPU and suicide rates, but there appears to be no association between the two. Tables A5–A8 present results by population density *and* age group and occupation, respectively. Results are similar to those presented in Tables 2 and 3 The magnitude for self-employed people is particularly large in high-population density areas, while for unemployed people it is high in less-density areas. We also ran the same regressions aggregating at the quarterly level. Results are largely the same and hold the same interpretation (Table A9). Results are also robust to excluding unemployment as control variable (Table A10).

## Discussion

This paper studied the association between economic uncertainty and suicide in Japan, providing evidence by sex, occupation, and population density. We found that a 1% increase in economic uncertainty is associated with a 0.061 increase in the monthly number of suicides per 100,000 people per prefecture, on average (coefficient: 6.08; 95% CI: 5.07–7.08); which constitutes a 3.62% increase. The magnitude of the association is about three times larger for males than females and is greatest for men in their 50s. In terms of working status, unemployed and self-employed people appear to be associated the most by economic uncertainty. The association is particularly strong for unemployed males in less-density areas and self-employed males in high-population density areas. Our findings add to a recent yet growing body of literature that has shown that economic uncertainty is associated with suicide.^18^, ^19^, ^20^, ^21^, ^22^, ^23^ This study contributes to the literature by studying differences by population group and adding the perspective of age, employment status, and regional differences. In addition, this is the first study that investigates the association of economic uncertainty with a specific emphasis on Japan. Our findings differ from previous studies that showed no correlation between the global EPU index and East Asian suicide rates,^22^ which emphasises the importance of studies using country-level EPUs, as not all countries demonstrate the same response to uncertainty.

Economic uncertainty is not the sole cause of suicide as overall low R^2^ values indicated. However, this study provides new insights into the understanding of suicide in Japan by age group, sex, employment status, and degree of population density. Our results can help understand which groups of people should be prioritised during times of increased economic uncertainty. While it is widely believed that suicide rates are higher among unemployed and older males during economic downturns^,^^9^^,^^21^^,^^43^ the higher association between uncertainty and suicide among the self-employed is a novel finding. This is not a surprising result, as their income is not fixed and depends on economic activity in their industry. The lack of safety nets such as unemployment insurance for the self-employed may also exacerbate their anxiety in turbulent periods. Japanese employment insurance, which provides unemployment benefits, is a system that allows unemployed people to receive between 50% and 80% of their last salary for 90–360 days, depending on their age, the insured period and reason for unemployment, but self-employed individuals are not eligible for this insurance.^44^ The median survival length from exposure to the cause of suicide to actual suicide is 3.5 years shorter among male self-employed founding directors compared to the general Japanese population.^45^ Introducing safety nets may provide much-needed support particularly to self-employed people who appear to experience the highest increase in risk in times of economic uncertainty. Strengthening mental health support, such as suicide hotlines, during periods of economic instability may also be effective in preventing suicide.

The association between uncertainty and suicides among unemployed people is also high, possibly because economic turbulence may affect the chances of getting a job. Mental health support, out-of-work benefits, and labour market training are particularly important for people who lose their jobs, especially during times of increased uncertainty. Economic uncertainty has been demonstrated to be associated with unhealthy lifestyles^46^ and poor subjective health,^29^ which may explain the link with increased suicide. One plausible factor that might contribute to the association between uncertainty and suicide, particularly among Japanese males might be traced to various issues attributed to overwork. Suicide due to overwork is referred to as *karōjisatsu*, where long working hours cause anxiety and health problems, which may in turn induce suicide.^47^^,^^48^ Examining the incident rates of cases involving worker's compensation for occupational suicide shows that approximately 95% of cases concerned male workers.^49^ Workloads increased in Japan after recessions, and economic uncertainty may make people work longer hours in order to be financially protected ahead of any anticipated financial turbulence.^50^ The lack of correlation between economic uncertainty and teenagers' suicides might also suggest that the link between economic uncertainty and suicide is closely tied to employment. Alternatively, young people might be more likely to wait for economic conditions to improve.^51^

There are several limitations of this study. Observations with one or two monthly suicides were not reported by the data source, to prevent any individual from being identified when combining with other sources of information. Moreover, unemployment rates are estimated by using quarterly prefectural averages and the calculation of the occupational suicide rate is based on a linear extrapolation of the population by occupation from the census that takes place every five years. Additionally, the data provided did not allow us to examine differences depending on skills or education level. It is also worth mentioning that data were available at the prefecture level, and population density was not uniformly distributed within each area. Even within high-density prefectures, there are rural areas, so our results by population density reflect the average of such areas and do not provide detailed information at the very small local level. It also should be recognised that the data used in this analysis are up to 2019, before the onset of the Covid-19 pandemic. Furthermore, our findings may be related to features specific to the Japanese working culture, which may limit generalisability beyond the Japan-specific cultural and social context. Finally, this paper presents evidence on the association between uncertainty and suicide, as did previous studies in this area,^18^, ^19^, ^20^, ^21^, ^22^, ^23^ but does not provide evidence of causality. Designing a study that addresses causality on this topic is particularly challenging, given the absence of any clear natural experiment. Future research can seek such settings in order to advance the literature in this area.

Overall, our paper suggests that there is a positive association between economic uncertainty and suicide in Japan and is the first to highlight the groups that experience the greatest increase in risk of suicide in periods of economic uncertainty.

## Contributors

HG, SV and IK conceived and designed the study. HG and SV conducted the literature review. HG extracted the data and performed the statistical analysis. HG and SV interpreted the findings. HG and SV drafted the article. IK, SV and HG critically revised it for important intellectual content.

## Data sharing statement

The data referenced in this article are publicly accessible and cited in the reference list. Data can be provided upon reasonable request.

## Declaration of interests

The authors declare that there are no conflicts of interest to disclose.

## Appendix A Supplementary data

Abstract in Japanese

## References

[1] World Health Organisation (2022). https://www.who.int/newsroom/fact-sheets/detail/suicide.

[2] Ministry of Health, Labour and Welfare (2022). https://www.mhlw.go.jp/stf/seisakunitsuite/bunya/hukushi_kaigo/seikatsuhogo/jisatsu/jisatsuhakusyo2022.html.

[3] World Health Organisation (2021).

[4] Ministry of Health, Labour and Welfare (2022). https://www.mhlw.go.jp/wp/yosan/yosan/22syokan/.

[5] Department of Health & Social Care (2023). https://www.gov.uk/government/publications/suicide-prevention-strategy-for-england-2023-to-2028/suicide-prevention-in-england-5-year-cross-sector-strategy.

[6] Centers for Disease Control and Prevention (2023). https://www.cdc.gov/injury/budget/suicidepreventionpolicy/SuicidePreventionInvestment.html.

[7] Ruhm C.J. (2000). Are recessions good for your health?. Q J Econ.

[8] Reeves A., Stuckler D., McKee M., Gunnell D., Chang S.S., Basu S. (2012). Increase in state suicide rates in the USA during economic recession. Lancet.

[9] Gunnell D., Chang S.S. (2016).

[10] Huikari S., Korhonen M. (2021). Unemployment, global economic crises and suicides: evidence from 21 OECD countries. Appl Econ.

[11] Huikari S., Miettunen J., Korhonen M. (2019). Economic crises and suicides between 1970 and 2011: time trend study in 21 developed countries. J Epidemiol Community.

[12] Chen J., Choi Y.C., Mori K., Sawada Y., Sugano S. (2012). Recession, unemployment, and suicide in Japan. Japan Labor Review.

[13] Okada M., Hasegawa T., Kato R., Shiroyama T. (2020). Analysing regional unemployment rates, GDP per capita and financial support for regional suicide prevention programme on suicide mortality in Japan using governmental statistical data. BMJ Open.

[14] Laliotis I., Ioannidis J.P., Stavropoulou C. (2016). Total and cause-specific mortality before and after the onset of the Greek economic crisis: an interrupted time-series analysis. Lancet Public Health.

[15] Kawachi I., Kyriopoulos I., Vandoros S. (2023). Economic uncertainty and cardiovascular disease mortality. Health Econ.

[16] Chang T., Chen W.Y. (2017). Revisiting the relationship between suicide and unemployment: evidence from linear and nonlinear cointegration. Econ Syst.

[17] Arbatli-Saxegaard E.C.A., Davis S.J., Ito A., Miake N. (2022). Policy uncertainty in Japan. J Jpn Int Econ.

[18] Antonakakis N., Gupta R. (2017). Is economic policy uncertainty related to suicide rates? Evidence from the United States. Soc Indicat Res.

[19] Vandoros S., Avendano M., Kawachi I. (2019). The association between economic uncertainty and suicide in the short-run. Soc Sci Med.

[20] Vandoros S., Kawachi I. (2021). Economic uncertainty and suicide in the United States. Eur J Epidemiol.

[21] Abdou R., Cassells D., Berrill J., Hanly J. (2022). Revisiting the relationship between economic uncertainty and suicide: an alternative approach. Soc Sci Med.

[22] Claveria O. (2022). Global economic uncertainty and suicide: worldwide evidence. Soc Sci Med.

[23] Er S.T., Ender D., Emre S. (2023). Suicide and economic uncertainty: new findings in a global setting. SSM Popul Health.

[24] Kyriopoulos I., Vandoros S., Kawachi I. (2023). State-level economic uncertainty and cardiovascular disease deaths: evidence from the United States. Eur J Epidemiol.

[25] Vandoros S., Avendano M., Kawachi I. (2018). The short-term impact of economic uncertainty on motor vehicle collisions. Prev Med.

[26] Kavetsos G., Kawachi I., Kyriopoulos I., Vandoros S. (2021). The effect of the Brexit referendum result on subjective well-being. J Roy Stat Soc.

[27] Vandoros S., Avendano M., Kawachi I. (2019). The EU referendum and mental health in the short term: a natural experiment using antidepressant prescriptions in England. J Epidemiol Community Health.

[28] Metcalfe R., Powdthavee N., Dolan P. (2011). Destruction and distress: using a quasi-experiment to show the effects of the September 11 attacks on mental well-being in the United Kingdom. Econ J.

[29] Tao H.L., Cheng H.P. (2023). Economic policy uncertainty and subjective health: a gender perspective. Soc Sci Med.

[30] Min J.Y., Kim H., Park S.G., Hwang S.H., Min K.B. (2019). Differences in suicidal behaviors between self-employed and standardly employed workers. Am J Ind Med.

[31] Middleton N., Gunnell D., Frankel S., Whitley E., Dorling D. (2003). Urban–rural differences in suicide trends in young adults: England and Wales, 1981–1998. Soc Sci Med.

[32] Liu L. (2023). Economic uncertainty and population health: insights from emerging markets and developing countries. Front Public Health.

[33] Basic Data on Suicide in the Region (BDSR) Table A5. https://www.mhlw.go.jp/stf/seisakunitsuite/bunya/0000140901.html.Japanese.

[34] Kyriopoulos I., Vandoros S., Kawachi I. (2022). Police killings and suicide among Black Americans. Soc Sci Med.

[35] Population Estimate II. Population as of 1 October in each year. Prefectures. https://www.stat.go.jp/data/jinsui/2.html.Japanese.

[36] National Census Basic aggregation of employment status. 2005, 2010, 2015, 2020. https://www.e-stat.go.jp/stat-search/database?page=1&toukei=00200521.Japanese.

[37] Basic school Survey. https://www.e-stat.go.jp/stat-search/files?page=1&toukei=00400001&tstat=000001011528.Japanese.

[38] Economic Policy Uncertainty (2022). https://www.policyuncertainty.com/japan_monthly.html.

[39] Baker S.R., Bloom N., Davis S.J. (2016). Measuring economic policy uncertainty. Q J Econ.

[40] Labour Force Survey Results by prefecture (model estimates). Quarterly average. https://www.e-stat.go.jp/stat-search/files?page=1&toukei=00200531&tstat=000000110001&tclass1=000001011635.Japanese.

[41] Helbich M., Blueml V., de Jong T., Plener P.L., Kwan M.P., Kapusta N.D. (2017). Urban–rural inequalities in suicide mortality: a comparison of urbanicity indicators. Int J Health Geogr.

[42] Verbeek M. (2008).

[43] Mäki N., Martikainen P. (2012). A register-based study on excess suicide mortality among unemployed men and women during different levels of unemployment in Finland. J Epidemiol Community.

[44] Ministry of Health Labour and Welfare (2022). https://www.mhlw.go.jp/stf/seisakunitsuite/bunya/0000134526.html.

[45] Sakisaka K. (2018). Identification of high risk groups with shorter survival times after onset of the main reason for suicide: findings from interviews with the bereaved in Japan. BMC Res Notes.

[46] Kalcheva I., McLemore P., Sias R. (2021). Economic policy uncertainty and self-control: evidence from unhealthy choices. J Financ Quant Anal.

[47] Bannai A., Tamakoshi A. (2014). The association between long working hours and health: a systematic review of epidemiological evidence. Scand J Work Environ Health.

[48] Virtanen M., Ferrie J.E., Singh-Manoux A. (2011). Long working hours and symptoms of anxiety and depression: a 5-year follow-up of the Whitehall II study. Psychol Med.

[49] Yamauchi T., Sasaki T., Yoshikawa T., Matsumoto S., Takahashi M. (2018). Incidence of overwork-related mental disorders and suicide in Japan. Occup Med.

[50] Kondo N., Oh J. (2010). Suicide and karoshi (death from overwork) during the recent economic crises in Japan: the impacts, mechanisms and political responses. J Epidemiol Community.

[51] Cutler D.M., Glaeser E.L., Norberg K.E., Gruber J. (2001). Risky behavior among youths*: an economic analysis*.

