# Supplementary material for: The association between economic uncertainty and suicide in Japan by age, sex, employment status, and population density: an observational study
Source: Lancet Reg Health West Pac. 2024 May 2;46:101069. doi: 10.1016/j.lanwpc.2024.101069 (PMC11070334; doi:10.1016/j.lanwpc.2024.101069)
Supplement: Abstract in Japanese [file mmc1.docx]

*This translation in Japanese was submitted by the authors and we reproduce it as supplied. It has not been peer reviewed. Our editorial processes have only been applied to the original abstract in English, which should serve as reference for this manuscript*.

概要

背景

自殺は世界10大死因の1つであり、先行研究では経済状況とメンタルヘルスの関連が指摘されている。しかしこれらの文献は主に不況や失業、すなわち実際の経済動向に焦点を当てており、経済動向の不確実性との関係はわかっていない。本研究では、性別、年齢、雇用形態、人口密度によって異なる、日本における経済不確実性と自殺の関連を検討し、最もリスクの高い集団を特定することを目的とする。

方法

2009年から2019年までの厚生労働省の月別・都道府県別の自殺死亡データと月別経済不確実性指数を用い、固定効果パネルデータ法を用いて不確実性と自殺死亡率（人口10万人当たり自殺者数）の関連を人口集団別に検討した。

結果

経済不確実性が1％上昇すると、月別・都道府県別の人口10万人当たり自殺者数が0.061（係数：6.08、95％信頼区間：5.07-7.08）人増加し、3.62％増加することが観察された。特に自営業者、50歳代男性、無職男性の自殺死亡率は経済不確実性と強く関連していることがわかった。また、この関連は女性よりも男性で約3倍強く、人口密度の高い地域に住む自営業の男性で強く見られた。

解釈

経済不確実性はほとんどの集団で自殺に関連しているようであるが、特に自営業者、男性、人口密度の高い地域に住む人々は、経済的不確実性が高まる時期に自殺リスクが高まるようである。我々の結果は、不確実性の高い時代のメンタルヘルスサービスや自殺予防戦略においてどの集団を優先すべきかを示すものである。
