# Supplementary material for: The association between economic uncertainty and suicide in Japan by age, sex, employment status, and population density: an observational study
Source: Lancet Reg Health West Pac. 2024 May 2;46:101069. doi: 10.1016/j.lanwpc.2024.101069 (PMC11070334; doi:10.1016/j.lanwpc.2024.101069)
Supplement: Supplementary Tables [file mmc2.pdf]

ONLINE APPENDIX

Table A1: Correlation between the unemployment rate and the EPU

Dependent Variable: Economic Policy Uncertainty

	**Regression model**		**Lagged regression model**	
	**EPU**	**ln EPU**	**Lagged EPU**	**Lagged ln EPU**
	(1)	(2)	(3)	(4)
**Unemployment rate**	5.95**	0.05**	6.06**	0.05***
	(0.82 - 11.08)	(0.01 - 0.10)	(0.91 - 11.21)	(0.01 - 0.10)
**Constant**	95.95***	4.54***	95.79***	4.54***
	(77.02 - 114.87)	(4.39 - 4.69)	(76.82 - 114.76)	(4.39 - 4.69)
**Observations**	156	156	155	155
**Adjusted R-squared**	0.027	0.035	0.028	0.037
**F-value**	5.253	6.692	5.396	6.869
**Degrees of Freedom**	154	154	153	153

Confidence intervals in parentheses. *** p<0.01, ** p<0.05, * p<0.1

Table A2: Summary Statistics

	All prefectures (n=47)					
	Total		Male		Female	
	Mean	SD	Mean	SD	Mean	SD
**Total**						
**N of suicides per month**	41.97	41.24	28.84	27.76	13.14	14.10
**Population (1,000s)**	2707.2	2691.49	1317.32	1330.73	1389.86	1361.64
**Monthly suicide rate per 100,000 people**	1.68	0.52	2.43	0.84	0.98	0.41
**Self-employed individuals**						
**N of suicides per month**	3.22	3.33	3.01	3.08	0.39	0.69
**Population (1,000s)**	155.98	113.34	94.73	69.95	61.25	43.6
**Monthly suicide rate per 100,000 people**	2.19	1.56	3.26	2.38	0.65	1.21
**Employed individuals**						
**N of suicides per month**	11.89	12.02	10.28	9.93	2.31	2.86
**Population (1,000s)**	1055.91	1024.4	585.73	580.13	470.18	445.44
**Monthly suicide rate per 100,000 people**	1.21	0.53	1.85	0.85	0.47	0.43
**Unemployed individuals**						
**N of suicides per month**	1.86	2.49	1.77	2.34	0.21	0.50
**Population (1,000s)**	65.66	65.07	42.99	42.27	22.67	22.92
**Monthly suicide rate per 100,000 people**	3.21	3.5	4.4	4.79	1.02	3.02
**Students**						
**N of suicides per month**	1.50	2.21	1.11	1.64	0.49	0.95
**Population (1,000s)**	340.2	354.19	176.4	184.57	163.8	169.69
**Monthly suicide rate per 100,000 people**	0.42	0.48	0.58	0.76	0.25	0.52
**Economic policy uncertainty**	116.96	29.65	116.96	29.65	116.96	29.65
**Unemployment rate (%)**	3.40	1.19	3.40	1.19	3.40	1.19

Table A2: Summary Statistics (continued)

	Prefectures in higher-density areas (n=8)						Prefectures in lower-density areas (n=39)					
	Total		Male		Female		Total		Male		Female	
	Mean	SD	Mean	SD	Mean	SD	Mean	SD	Mean	SD	Mean	SD
**Total**												
**N of suicides per month**	119.05	45.15	80.60	30.68	38.45	16.00	28.03	17.81	19.59	12.63	8.44	5.97
**Population (1,000s)**	7872.88	2503.93	3870.99	1257.12	4001.89	1250.37	1647.57	920.59	793.48	444.78	854.06	476.5
**Monthly suicide rate per 100,000 people**	1.53	0.35	2.12	0.54	0.96	0.25	1.71	0.54	2.50	0.88	0.98	0.44
**Self-employed individuals**												
**N of suicides per month**	7.93	4.51	7.13	4.13	0.80	0.93	2.55	2.28	2.36	2.12	0.32	0.60
**Population (1,000s)**	362.5	100.6	224.03	61.12	138.47	40.26	113.62	53.49	68.21	31.58	45.41	22.01
**Monthly suicide rate per 100,000 people**	2.19	1.06	3.19	1.6	0.58	0.65	2.19	1.64	3.28	2.53	0.67	1.30
**Employed individuals**												
**N of suicides per month**	32.3	13.26	26.42	10.92	5.85	3.56	8.04	5.92	7.04	5.14	1.43	1.50
**Population (1,000s)**	3039.14	872.54	1708.75	497.24	1330.39	382.25	649.1	368.97	355.37	207.59	293.73	161.92
**Monthly suicide rate per 100,000 people**	1.08	0.32	1.57	0.48	0.44	0.22	1.23	0.56	1.91	0.91	0.47	0.46
**Unemployed individuals**												
**N of suicides per month**	5.00	3.86	4.49	3.56	0.50	0.78	1.42	1.75	1.35	1.67	0.15	0.41
**Population (1,000s)**	186.41	63.46	121.14	41.78	65.27	22.18	40.89	25.93	26.96	16.88	13.93	9.14
**Monthly suicide rate per 100,000 people**	2.59	1.65	3.56	2.34	0.75	1.15	3.34	3.75	4.59	5.17	1.07	3.29
**Students**												
**N of suicides per month**	4.57	3.09	3.23	2.34	1.34	1.39	0.85	1.06	0.64	0.88	0.25	0.53
**Population (1,000s)**	1011.17	366.37	525.88	191.67	485.29	175.08	202.56	111.17	104.71	57.69	97.85	53.51
**Monthly suicide rate per 100,000 people**	0.45	0.25	0.62	0.39	0.27	0.27	0.41	0.51	0.58	0.82	0.25	0.56
**Economic policy uncertainty**	116.96	29.66	116.96	29.66	116.96	29.66	116.96	29.65	116.96	29.65	116.96	29.65
**Unemployment rate (%)**	3.81	1.16	3.81	1.16	3.81	1.16	3.31	1.18	3.31	1.18	3.31	1.18

Table A3: Results of the *Interaction model* by sex and population density

Dependent Variable: Suicides per 100,000 people									
	**Whole**			**Urban**			**Rural**		
	**Total**	**Male**	**Female**	**Total**	**Male**	**Female**	**Total**	**Male**	**Female**
	(1)	(2)	(3)	(4)	(5)	(6)	(7)	(8)	(9)
**Uncertainty**	5.93***	9.09***	3.01***	4.73***	7.02***	2.37***	6.02***	9.26***	3.07***
	(4.91 - 6.95)	(7.32 - 10.86)	(1.98 - 4.03)	(3.55 - 5.91)	(5.06 - 8.98)	(1.16 - 3.58)	(4.82 - 7.23)	(7.17 - 11.36)	(1.86 - 4.28)
**Unemployment rate**	-0.20	-0.37	-0.04	-0.33*	-0.41	-0.26	-0.26	-0.51*	-0.02
	(-0.46 - 0.06)	(-0.83 - 0.08)	(-0.30 - 0.22)	(-0.69 - 0.03)	(-1.01 - 0.18)	(-0.63 - 0.10)	(-0.57 - 0.05)	(-1.05 - 0.03)	(-0.34 - 0.29)
**Interaction between uncertainty and unemployment rate**	0.05*	0.09*	0.01	0.08**	0.11*	0.06	0.06*	0.11**	0.01
	(-0.01 - 0.10)	(-0.01 - 0.18)	(-0.04 - 0.06)	(0.01 - 0.16)	(-0.01 - 0.23)	(-0.02 - 0.14)	(-0.01 - 0.12)	(0.00 - 0.23)	(-0.06 - 0.07)
**Constant**	-26.86***	-41.30***	-13.54***	-21.39***	-31.95***	-10.49***	-27.30***	-42.07***	-13.86***
	(-31.73 - -22.00)	(-49.73 - -32.87)	(-18.43 - -8.66)	(-26.98 - -15.79)	(-41.27 - -22.63)	(-16.24 - -4.74)	(-33.06 - -21.54)	(-52.06 - -32.08)	(-19.64 - -8.08)
**Observations**	6,204	6,204	6,204	1,056	1,056	1,056	5,148	5,148	5,148
**N of Prefectures**	47	47	47	8	8	8	39	39	39
**Adjusted R-squared**	0.466	0.396	0.207	0.773	0.717	0.537	0.440	0.374	0.186
**F-value**	42.05	31.88	13.56	28.02	21.13	10.25	31.64	24.37	10.15
**Degrees of Freedom**	6,024	6,024	6,024	915	915	915	4,976	4,976	4,976

Notes: Fixed Effects Panel Data. Uncertainty in natural logarithm. Year-month dummies included. Confidence intervals in parentheses. *** p<0.01, ** p<0.05, * p<0.1

Table A4: Results of the *lagged model* by sex and population density

Dependent Variable: Suicides per 100,000 people

	Whole			Urban			Rural		
	**Total**	**Male**	**Female**	**Total**	**Male**	**Female**	**Total**	**Male**	**Female**
	(1)	(2)	(3)	(4)	(5)	(6)	(7)	(8)	(9)
**Uncertainty**	2.17***	3.03***	1.37***	1.37***	1.99**	0.75	2.31***	3.20***	1.49***
	(1.33 - 3.00)	(1.59 - 4.47)	(0.54 - 2.21)	(0.43 - 2.31)	(0.43 - 3.55)	(-0.21 - 1.71)	(1.32 - 3.29)	(1.50 - 4.91)	(0.50 - 2.48)
**lagged uncertainty**	-1.32	-0.88	-1.75	-0.32	-0.36	-0.40	-1.55	-1.02	-2.03
	(-3.61 - 0.96)	(-4.84 - 3.08)	(-4.05 - 0.55)	(-2.89 - 2.25)	(-4.63 - 3.91)	(-3.03 - 2.23)	(-4.25 - 1.15)	(-5.70 - 3.66)	(-4.75 - 0.69)
**Unemployment rate**	0.02	0.03	0.01	0.07***	0.12***	0.02	0.02	0.03	0.01
	(-0.01 - 0.04)	(-0.01 - 0.08)	(-0.02 - 0.03)	(0.03 - 0.10)	(0.06 - 0.18)	(-0.02 - 0.06)	(-0.01 - 0.04)	(-0.02 - 0.08)	(-0.02 - 0.04)
**Constant**	-2.81	-8.50	2.49	-3.97	-6.47	-0.93	-2.37	-8.57	3.28
	(-10.26 - 4.64)	(-21.41 - 4.41)	(-5.01 - 10.00)	(-12.36 - 4.43)	(-20.43 - 7.50)	(-9.53 - 7.67)	(-11.17 - 6.43)	(-23.84 - 6.70)	(-5.58 - 12.14)
**Observations**	6,157	6,157	6,157	1,048	1,048	1,048	5,109	5,109	5,109
**N of Prefectures**	47	47	47	8	8	8	39	39	39
**Adjusted R-squared**	0.464	0.393	0.207	0.768	0.712	0.534	0.438	0.371	0.186
**F-value**	42.05	31.79	13.63	27.58	20.80	10.21	31.68	24.31	10.22
**Degrees of Freedom**	5,979	5,979	5,979	909	909	909	4,939	4,939	4,939

Notes: Fixed Effects Panel Data. Uncertainty in natural logarithm. Year-month dummies included. Confidence intervals in parentheses. *** p<0.01, ** p<0.05, * p<0.1

Table A5: Results by Age-group *in high-density areas*

Dependent Variable: Suicides per 100,000 people	10-19			20-29			30-39			40-49		
	**Total**	**Male**	**Female**	**Total**	**Male**	**Female**	**Total**	**Male**	**Female**	**Total**	**Male**	**Female**
	(1)	(2)	(3)	(4)	(5)	(6)	(7)	(8)	(9)	(10)	(11)	(12)
**Uncertainty**	0.12	-0.26	0.50	5.27***	8.32***	1.91	4.10***	3.93	4.39**	7.02***	12.53***	1.05
	(-0.78 - 1.01)	(-1.70 - 1.19)	(-0.59 - 1.59)	(1.97 - 8.56)	(3.00 - 13.65)	(-1.70 - 5.53)	(1.07 - 7.14)	(-0.94 - 8.81)	(1.03 - 7.75)	(3.94 - 10.10)	(7.42 - 17.65)	(-2.18 - 4.28)
**Unemployment rate**	-0.00	-0.00	-0.00	0.05	0.02	0.08	0.05	0.07	0.04	0.09*	0.14*	0.05
	(-0.03 - 0.03)	(-0.05 - 0.04)	(-0.04 - 0.03)	(-0.06 - 0.15)	(-0.15 - 0.19)	(-0.03 - 0.19)	(-0.04 - 0.15)	(-0.09 - 0.22)	(-0.06 - 0.15)	(-0.01 - 0.18)	(-0.02 - 0.30)	(-0.06 - 0.15)
**Constant**	-0.38	1.50	-2.34	-23.93***	-38.23***	-8.26	-18.06**	-16.66	-20.05**	-32.15***	-57.99***	-4.21
	(-4.65 - 3.88)	(-5.37 - 8.37)	(-7.54 - 2.87)	(-39.59 - - 8.27)	(-63.55 - - 12.92)	(-25.45 - 8.92)	(-32.50 - - 3.63)	(-39.85 - 6.52)	(-36.03 - - 4.06)	(-46.81 - - 17.50)	(-82.31 - - 33.66)	(-19.58 - 11.16)
**Observations**	1,056	1,056	1,056	1,056	1,056	1,056	1,056	1,056	1,056	1,056	1,056	1,056
**N of Prefectures**	8	8	8	8	8	8	8	8	8	8	8	8
**Adjusted R-squared**	0.087	0.034	0.059	0.259	0.178	0.159	0.309	0.244	0.142	0.474	0.439	0.147
**F-value**	1.819	1.335	1.551	3.850	2.778	2.569	4.630	3.631	2.376	8.243	7.301	2.427
**Degrees of Freedom**	916	916	916	916	916	916	916	916	916	916	916	916

Notes: Fixed Effects Panel Data. Uncertainty in natural logarithm. Year-month dummies included. Confidence intervals in parentheses. *** p<0.01, ** p<0.05, * p<0.1

Table A5: Results by Age-group *in high-density areas* (continued)

Dependent Variable: Suicides per 100,000 people

	50-59			60-69			70-79			80-		
	**Total**	**Male**	**Female**	**Total**	**Male**	**Female**	**Total**	**Male**	**Female**	**Total**	**Male**	**Female**
	(13)	(14)	(15)	(16)	(17)	(18)	(19)	(20)	(21)	(22)	(23)	(24)
**Uncertainty**	9.31***	17.31***	1.24	8.39***	11.36***	5.50***	2.56	1.93	2.99	7.11***	9.05*	6.27**
	(5.64 - 12.98)	(11.07 - 23.56)	(-2.51 - 4.99)	(5.21 - 11.57)	(5.94 - 16.79)	(2.29 - 8.72)	(-1.14 - 6.25)	(-4.31 - 8.16)	(-1.18 - 7.16)	(2.37 - 11.86)	(-1.27 - 19.37)	(1.42 - 11.12)
**Unemployment rate**	0.09	0.18*	-0.00	0.07	0.16*	-0.01	0.20***	0.38***	0.06	0.01	0.19	-0.08
	(-0.03 - 0.20)	(-0.01 - 0.38)	(-0.12 - 0.12)	(-0.03 - 0.17)	(-0.01 - 0.33)	(-0.11 - 0.09)	(0.09 - 0.32)	(0.19 - 0.58)	(-0.08 - 0.19)	(-0.14 - 0.16)	(-0.13 - 0.51)	(-0.23 - 0.07)
**Constant**	-42.79***	-80.49***	-4.79	-38.55***	-52.35***	-25.18***	-11.16	-8.07	-13.35	-32.39***	-41.25*	-28.60**
	(-60.26 - - 25.32)	(-110.20 - - 50.78)	(-22.63 - 13.06)	(-53.69 - - 23.40)	(-78.17 - - 26.53)	(-40.49 - - 9.86)	(-28.74 - 6.41)	(-37.74 - 21.61)	(-33.18 - 6.48)	(-54.95 - - 9.84)	(-90.34 - 7.83)	(-51.69 - - 5.52)
**Observations**	1,056	1,056	1,056	1,056	1,056	1,056	1,056	1,056	1,056	1,056	1,056	1,056
**N of Prefectures**	8	8	8	8	8	8	8	8	8	8	8	8
**Adjusted R-squared**	0.456	0.451	0.086	0.547	0.496	0.234	0.303	0.215	0.140	0.179	0.074	0.147
**F-value**	7.764	7.620	1.807	10.70	8.904	3.492	4.522	3.248	2.349	2.794	1.691	2.425
**Degrees of Freedom**	916	916	916	916	916	916	916	916	916	916	916	916

Notes: Fixed Effects Panel Data. Uncertainty in natural logarithm. Year-month dummies included. Confidence intervals in parentheses. *** p<0.01, ** p<0.05, * p<0.1

Table A6: Results by Age-group *in low-density areas*

Dependent Variable: Suicides per 100,000 people	10-19			20-29			30-39			40-49		
	**Total**	**Male**	**Female**	**Total**	**Male**	**Female**	**Total**	**Male**	**Female**	**Total**	**Male**	**Female**
	(1)	(2)	(3)	(4)	(5)	(6)	(7)	(8)	(9)	(10)	(11)	(12)
**Uncertainty**	0.30	1.02	-0.47	6.29***	9.52***	2.74	5.91***	7.32**	4.66***	4.86***	7.03**	2.87*
	(-0.61 - 1.21)	(-0.44 - 2.48)	(-1.55 - 0.60)	(2.35 - 10.23)	(2.92 - 16.13)	(-1.30 - 6.77)	(2.47 - 9.35)	(1.37 - 13.28)	(1.26 - 8.06)	(1.38 - 8.33)	(0.88 - 13.19)	(-0.48 - 6.23)
**Unemployment rate**	0.01	-0.01	0.03**	-0.05	-0.14*	0.06	0.02	0.03	0.02	0.06	0.10	0.03
	(-0.02 - 0.03)	(-0.05 - 0.02)	(0.00 - 0.05)	(-0.14 - 0.05)	(-0.30 - 0.01)	(-0.03 - 0.16)	(-0.06 - 0.10)	(-0.11 - 0.17)	(-0.06 - 0.10)	(-0.02 - 0.15)	(-0.04 - 0.25)	(-0.05 - 0.11)
**Constant**	-1.26	-4.53	2.31	-28.40***	-43.05***	-12.28	-26.62***	-32.65**	-21.39***	-21.31**	-30.74**	-12.87
	(-5.59 - 3.08)	(-11.50 - 2.43)	(-2.82 - 7.43)	(-47.21 - - 9.58)	(-74.59 - - 11.51)	(-31.53 - 6.97)	(-43.04 - - 10.20)	(-61.07 - - 4.23)	(-37.61 - - 5.18)	(-37.89 - - 4.74)	(-60.11 - - 1.36)	(-28.88 - 3.15)
**Observations**	5,148	5,145	5,109	5,148	5,145	5,109	5,148	5,145	5,109	5,148	5,145	5,109
**N of Prefectures**	39	39	39	39	39	39	39	39	39	39	39	39
**Adjusted R-squared**	0.004	-0.002	-0.005	0.041	0.025	0.013	0.079	0.050	0.033	0.141	0.121	0.022
**F-value**	1.427	1.214	1.081	2.956	2.304	1.786	4.636	3.344	2.624	7.662	6.667	2.151
**Degrees of Freedom**	4,977	4,974	4,938	4,977	4,974	4,938	4,977	4,974	4,938	4,977	4,974	4,938

Notes: Fixed Effects Panel Data. Uncertainty in natural logarithm. Year-month dummies included. Confidence intervals in parentheses. *** p<0.01, ** p<0.05, * p<0.1

Table A6: Results by Age-group *in low-density areas* (continued)

Dependent Variable: Suicides per 100,000 people	50-59			60-69			70-79			80-		
	**Total**	**Male**	**Female**	**Total**	**Male**	**Female**	**Total**	**Male**	**Female**	**Total**	**Male**	**Female**
	(13)	(14)	(15)	(16)	(17)	(18)	(19)	(20)	(21)	(22)	(23)	(24)
**Uncertainty**	11.91***	20.96***	2.88	9.00***	12.18***	5.99***	6.67***	9.25***	4.80**	8.02***	19.10***	2.61
	(8.14 - 15.67)	(14.30 - 27.61)	(-0.78 - 6.54)	(5.73 - 12.26)	(6.53 - 17.83)	(2.59 - 9.40)	(2.91 - 10.44)	(2.59 - 15.91)	(0.66 - 8.94)	(3.65 - 12.39)	(9.44 - 28.77)	(-1.73 - 6.95)
**Unemployment rate**	0.08*	0.16**	0.00	0.04	0.11	-0.01	0.04	0.10	-0.00	-0.10*	-0.26**	-0.00
	(-0.01 - 0.17)	(0.01 - 0.32)	(-0.08 - 0.09)	(-0.03 - 0.12)	(-0.02 - 0.24)	(-0.09 - 0.07)	(-0.05 - 0.13)	(-0.06 - 0.26)	(-0.10 - 0.10)	(-0.20 - 0.00)	(-0.49 - - 0.04)	(-0.11 - 0.10)
**Constant**	-54.87***	-97.13***	-12.71	-41.40***	-55.80***	-27.83***	-30.48***	-42.31***	-21.96**	-36.28***	-87.53***	-11.34
	(-72.84 - - 36.91)	(-128.89 - - 65.37)	(-30.17 - 4.75)	(-56.97 - - 25.82)	(-82.76 - - 28.85)	(-44.08 - - 11.58)	(-48.44 - - 12.51)	(-74.09 - - 10.53)	(-41.71 - - 2.21)	(-57.14 - - 15.43)	(-133.65 - - 41.42)	(-32.06 - 9.38)
**Observations**	5,148	5,145	5,109	5,148	5,145	5,109	5,148	5,145	5,109	5,148	5,145	5,109
**N of Prefectures**	39	39	39	39	39	39	39	39	39	39	39	39
**Adjusted R-squared**	0.179	0.173	0.011	0.180	0.147	0.041	0.104	0.064	0.045	0.083	0.035	0.055
**F-value**	9.816	9.465	1.728	9.821	8.030	2.959	5.795	3.945	3.126	4.823	2.682	3.550
**Degrees of Freedom**	4,977	4,974	4,938	4,977	4,974	4,938	4,977	4,974	4,938	4,977	4,974	4,938

Notes: Fixed Effects Panel Data. Uncertainty in natural logarithm. Year-month dummies included. Confidence intervals in parentheses. *** p<0.01, ** p<0.05, * p<0.1

Table A7: Results by Occupation *in high-density areas*

Dependent Variable: Suicides per 100,000 people

	Self-employed			Employed			Unemployed			Student		
	Total	Male	Female	Total	Male	Female	Total	Male	Female	Total	Male	Female
	(1)	(2)	(3)	(4)	(5)	(6)	(7)	(8)	(9)	(10)	(11)	(12)
**Uncertainty**	13.66***	19.26***	4.77**	4.13***	5.55***	1.58**	16.97***	21.42***	5.92	1.76**	2.02	1.34
	(7.67 - 19.65)	(10.14 - 28.37)	(0.28 - 9.26)	(2.51 - 5.75)	(2.96 - 8.15)	(0.08 - 3.09)	(7.47 - 26.47)	(7.71 - 35.13)	(-2.13 - 13.98)	(0.16 - 3.36)	(-0.56 - 4.60)	(-0.46 - 3.14)
**Unemployment rate**	0.09	0.17	-0.05	0.07***	0.11***	0.02	-0.05	-0.09	0.03	0.00	0.01	0.00
	(-0.10 - 0.28)	(-0.12 - 0.45)	(-0.19 - 0.09)	(0.02 - 0.12)	(0.03 - 0.20)	(-0.03 - 0.07)	(-0.35 - 0.24)	(-0.52 - 0.34)	(-0.22 - 0.28)	(-0.05 - 0.05)	(-0.08 - 0.09)	(-0.05 - 0.06)
**Constant**	-63.89***	-89.99***	-22.43**	-18.80***	-25.22***	-7.12*	-77.80***	-97.77***	-27.39	-7.97**	-9.06	-6.15
	(-92.38 - -35.40)	(-133.33 - -46.65)	(-43.80 - -1.07)	(-26.51 - -11.09)	(-37.56 - -12.88)	(-14.28 - 0.04)	(-122.98 - -32.61)	(-162.98 - -32.55)	(-65.71 - 10.93)	(-15.57 - -0.37)	(-21.34 - 3.22)	(-14.73 - 2.42)
**Observations**	1,056	1,056	1,056	1,056	1,056	1,056	1,056	1,056	1,056	1,056	1,056	1,056
**N of Prefectures**	8	8	8	8	8	8	8	8	8	8	8	8
**Adjusted R-squared**	0.326	0.320	0.050	0.454	0.385	0.080	0.311	0.291	0.019	0.115	0.070	0.073
**F-value**	4.922	4.806	1.475	7.699	6.056	1.751	4.665	4.329	1.211	2.092	1.651	1.679
**Degrees of Freedom**	916	916	916	916	916	916	916	916	916	916	916	916

Notes: Fixed Effects Panel Data. Uncertainty in natural logarithm. Year-month dummies included. Confidence intervals in parentheses. *** p<0.01, ** p<0.05, * p<0.1

Table A8: Results by Occupation *in low-density areas*

Dependent Variable: Suicides per 100,000 people												
	**Self-employed**			**Employed**			**Unemployed**			**Student**		
	**Total**	**Male**	**Female**	**Total**	**Male**	**Female**	**Total**	**Male**	**Female**	**Total**	**Male**	**Female**
	(1)	(2)	(3)	(4)	(5)	(6)	(7)	(8)	(9)	(10)	(11)	(12)
**Uncertainty**	8.42***	12.41***	5.16**	3.50***	5.02***	0.46	26.79***	31.40***	3.08	0.82	1.12	-0.06
	(3.65 - 13.20)	(4.53 - 20.29)	(0.78 - 9.54)	(1.94 - 5.07)	(2.27 - 7.76)	(-1.08 - 2.00)	(15.61 - 37.96)	(14.87 - 47.93)	(-7.90 - 14.05)	(-0.77 - 2.41)	(-1.61 - 3.85)	(-1.94 - 1.82)
**Unemployment rate**	0.04	-0.01	0.01	0.04**	0.07**	0.02	-0.36***	-0.36*	-0.07	-0.00	0.00	0.02
	(-0.08 - 0.15)	(-0.19 - 0.18)	(-0.10 - 0.11)	(0.00 - 0.07)	(0.01 - 0.14)	(-0.02 - 0.05)	(-0.62 - - 0.09)	(-0.74 - 0.02)	(-0.33 - 0.18)	(-0.04 - 0.04)	(-0.06 - 0.06)	(-0.03 - 0.06)
**Constant**	-38.34***	-56.28***	-24.28**	-15.58***	-22.24***	-1.76	-123.88***	-144.98***	-12.39	-3.52	-4.78	0.44
	(-61.13 - - 15.55)	(-93.96 - - 18.60)	(-45.22 - - 3.34)	(-23.05 - - 8.11)	(-35.37 - - 9.10)	(-9.11 - 5.59)	(-177.21 - - 70.55)	(-224.01 - - 65.95)	(-64.86 - 40.08)	(-11.11 - 4.07)	(-17.82 - 8.26)	(-8.56 - 9.43)
**Observations**	5,140	4,704	4,703	5,140	4,704	4,703	5,140	4,704	4,703	5,140	4,704	4,703
**N of Prefectures**	39	39	39	39	39	39	39	39	39	39	39	39
**Adjusted R-squared**	0.108	0.112	-0.007	0.160	0.142	0.018	0.056	0.055	0.001	0.006	0.004	-0.003
**F-value**	6.017	5.773	1.029	8.696	7.204	1.929	3.598	3.362	1.306	1.542	1.422	1.170
**Degrees of Freedom**	4,969	4,533	4,532	4,969	4,533	4,532	4,969	4,533	4,532	4,969	4,533	4,532

Notes: Fixed Effects Panel Data. Uncertainty in natural logarithm. Year-month dummies included. Confidence intervals in parentheses. *** p<0.01, ** p<0.05, * p<0.1

Table A9: Results of the baseline model *using quarterly data* by sex and population density

Dependent Variable: Suicides per 100,000 people									
	**Whole**			**Urban**			**Rural**		
	**Total**	**Male**	**Female**	**Total**	**Male**	**Female**	**Total**	**Male**	**Female**
	(1)	(2)	(3)	(4)	(5)	(6)	(7)	(8)	(9)
**Uncertainty**	4.07***	3.06***	4.22***	6.34***	4.68***	6.59***	1.96***	1.39***	2.05***
	(3.61 - 4.52)	(2.49 - 3.62)	(3.68 - 4.76)	(5.55 - 7.13)	(3.79 - 5.58)	(5.66 - 7.52)	(1.51 - 2.41)	(0.85 - 1.93)	(1.52 - 2.59)
**Unemployment rate**	0.02	0.07***	0.02	0.03	0.12***	0.03	0.01	0.02	0.01
	(-0.01 - 0.05)	(0.03 - 0.11)	(-0.01 - 0.05)	(-0.01 - 0.08)	(0.06 - 0.18)	(-0.02 - 0.08)	(-0.02 - 0.03)	(-0.01 - 0.06)	(-0.02 - 0.04)
**Constant**	-18.07***	-13.49***	-18.76***	-28.31***	-20.91***	-29.43***	-8.58***	-5.87***	-9.02***
	(-20.23 - -15.91)	(-16.15 - -10.84)	(-21.31 - -16.21)	(-32.05 - -24.58)	(-25.13 - -16.69)	(-33.86 - -25.01)	(-10.74 - -6.43)	(-8.41 - -3.33)	(-11.57 - -6.47)
**Observations**	2,068	352	1,716	2,068	352	1,716	2,068	352	1,716
**N of Prefectures**	47	8	39	47	8	39	47	8	39
**Adjusted R-squared**	0.697	0.891	0.675	0.633	0.871	0.611	0.414	0.756	0.383
**F-value**	110	66.27	82.66	82.99	55.23	62.99	35.19	25.93	26.04
**Degrees of Freedom**	1,977	300	1,633	1,977	300	1,633	1,977	300	1,633

Notes: Fixed Effects Panel Data. Uncertainty in natural logarithm. Year-month dummies included. Confidence intervals in parentheses. *** p<0.01, ** p<0.05, * p<0.1

Table A10: Results of the baseline model *without unemployment rate* by sex and population density

Dependent Variable: Suicides per 100,000 people									
	**Whole**			**Urban**			**Rural**		
	**Total**	**Male**	**Female**	**Total**	**Male**	**Female**	**Total**	**Male**	**Female**
	(1)	(2)	(3)	(4)	(5)	(6)	(7)	(8)	(9)
**Uncertainty**	6.42***	5.98***	6.51***	9.95***	9.12***	10.12***	3.18***	2.90***	3.23***
	(5.50 - 7.33)	(4.95 - 7.01)	(5.43 - 7.59)	(8.36 - 11.54)	(7.40 - 10.84)	(8.24 - 12.00)	(2.26 - 4.10)	(1.85 - 3.95)	(2.15 - 4.32)
**Constant**	-29.15***	-27.17***	-29.56***	-45.32***	-41.63***	-46.07***	-14.34***	-12.96***	-14.62***
	(-33.55 - -24.75)	(-32.12 - -22.21)	(-34.75 - -24.37)	(-52.94 - -37.70)	(-49.89 - -33.38)	(-55.09 - -37.06)	(-18.75 - -9.92)	(-18.01 - -7.91)	(-19.83 - -9.40)
**Observations**	6,204	1,056	5,148	6,204	1,056	5,148	6,204	1,056	5,148
**N of Prefectures**	47	8	39	47	8	39	47	8	39
**Adjusted R-squared**	0.466	0.769	0.439	0.395	0.712	0.373	0.208	0.536	0.187
**F-value**	42.62	27.83	32.08	32.30	20.95	24.69	13.76	10.36	10.31
**Degrees of Freedom**	6,026	917	4,978	6,026	917	4,978	6,026	917	4,978

Notes: Fixed Effects Panel Data. Uncertainty in natural logarithm. Year-month dummies included. Confidence intervals in parentheses. *** p<0.01, ** p<0.05, * p<0.1
